# The Intratumoral Heterogeneity Reflects the Intertumoral Subtypes of Glioblastoma Multiforme: A Regional Immunohistochemistry Analysis

**DOI:** 10.3389/fonc.2020.00494

**Published:** 2020-04-24

**Authors:** Natalie Bergmann, Claire Delbridge, Jens Gempt, Annette Feuchtinger, Axel Walch, Lucas Schirmer, Wolfram Bunk, Thomas Aschenbrenner, Friederike Liesche-Starnecker, Jürgen Schlegel

**Affiliations:** ^1^Division of Neuropathology, Technische Universität München, München, Germany; ^2^Institute of Pathology, Technische Universität München, München, Germany; ^3^Department of Neurosurgery, Technische Universität München, München, Germany; ^4^Research Unit Analytical Pathology, Helmholtz Zentrum München, German Research Center for Environmental Health (GmbH), Neuherberg, Germany; ^5^Department of Neurology, Universitätsklinikum Mannheim, Mannheim, Germany; ^6^Max-Planck-Institute for Extraterrestrial Physics, Garching, Germany

**Keywords:** glioblastoma, histological subtypes, intratumoral heterogeneity, intertumoral heterogeneity, histological architecture

## Abstract

Glioblastoma multiforme (GBM) is the most frequent and aggressive primary brain tumor in adults. Despite extensive therapy the prognosis for GBM patients remains poor and the extraordinary therapy resistance has been attributed to intertumoral heterogeneity of glioblastoma. Different prognostic relevant GBM tumor subtypes have been identified based on their molecular profile. This approach, however, neglects the heterogeneity within individual tumors, that is, the intratumoral heterogeneity. Here, we detected the regional immunoreactivity by immunohistochemistry and immunofluorescence using nine different markers on resected GBM specimens (IDH wildtype, WHO grade IV). We found repetitive expression profiles, that could be classified into clusters. These clusters could then be assigned to five pathophysiologically relevant groups that reflect the previously described subclasses of GBM, including mesenchymal, classical, and proneural subtype. Our data indicate the presence of tumor differentiations and tumor subclasses that occur within individual tumors, and might therefore contribute to develop adapted, individual-based therapies.

## Introduction

Glioblastoma is the most common malignant primary brain tumor, accounting for ~50% of all gliomas (1). The average survival rate is ~15 months (2) despite extensive therapy. For many years, various efforts failed to significantly improve the outcome. GBM remains a fatal disease with poor prognosis since relapse occurs regularly after resection, irradiation, and chemotherapy.

The morphological hallmark of glioblastoma is its heterogeneity, therefore the term “glioblastoma multiforme (GBM),” and it has been shown, that heterogeneity is an important reason why this aggressive neoplasm is so resistant to therapy. One of the most stimulating findings in recent years was the demonstration of different prognostic relevant subtypes using expression and transcriptome analyses (3–6). It illustrates the presence of heterogeneity in glioblastoma and tends to find a subgroup-orientated specific therapy according to the relevant molecular characteristics of each tumor subtype. However, this regime leads to an over-estimation of the intertumoral heterogeneity and neglected the additionally existing intratumoral heterogeneity (7). Within glioblastoma tissue many cells with different properties and levels of resistance to therapy are located. Consequently, recent therapy can only eliminate a fraction of the tumor cells, whereas others remain intact and finally cause the relapse. For fostering the development of more efficient antitumor therapies, it is necessary to conduct further investigations into the impact of intratumoral heterogeneity.

In this study, we therefore focused on regional intratumoral differences. We analyzed different areas within individual tumor samples with respect to their immunoreactivity for nine biomarkers relevant for the biology of GBM. We used a regional point of view to mirror the regional tumor characteristics, respecting the localization of the pathological transcription/translation and especially the intratumoral heterogeneity. Our results show that glioblastoma tissue can be classified into different clusters according to its respective immunoreactivity profile. These clusters can in a second step be assigned to larger pathophysiologically relevant groups and might therefore serve as targets for personalized treatment schedules.

## Materials and Methods

### Patient Collective

Human GBM tissue of 61 patients (30 female; 31 male) was included. All tumors were diagnosed as glioblastoma, IDH-wildtype, WHO grade IV according to the WHO-classification (8) by two neuropathologists (CD and JS). Neoplastic material of 53 patients (28 female; 25 male) was newly diagnosed GBM. Eight tumors were relapses and tissue consequently pretreated conventionally. The average patient age at the time of diagnosis was 60 years. The study has been evaluated and confirmed by Ethical Committee of the Technical University Munich.

### Immunohistochemistry

For immunostaining on paraffin-embedded and formalin-fixed human tumor tissue was cut into serial sections of 2 μm slides using a standard microtome.

Immunohistochemical staining was performed on a fully automated slide staining system (Ventana BenchMark ULTRA, Ventana Medical Systems, Tucson, USA) except for the detection of the target antigens ALDH1 and CA-IX that were manually processed. Briefly, after epitope unmasking (95°C in citrate buffer at pH 6.0) of deparaffinized slices, quenching of endogenous peroxidase (3% hydrogen peroxide solution for 15 min at room temperature) and blocking of non-specific binding sites (5% goat serum in Dako REAL™ antibody solution) slides were incubated with primary antibody (Table 1) at 4°C overnight, slides coated with Parafilm to minimize evaporation. Detection was performed with Dako REAL™ Detection System using biotinylated secondary antibody and subsequently with streptavidin peroxidase antibody (30 min). Staining was performed with DAB as chromogen (4 min) and counterstaining with Meyer's hemalaun. Positive controls were used as quality assurance.

**Table 1:** Primary antibodies.

**Target antigen**	**Species**	**Dilution (IHC)**	**Dilution (IF)**	**Provider**
ALDH1	Mouse	1:500	1:100	BD Transduction Laboratories (Heidelberg, Germany)
CA-IX	Rabbit	1:250	1:100	Cell Signaling Technology (Beverly, MA, USA)
EGFR	Mouse	1:50		Dako (Glostrup, Denmark)
FABP7	Rabbit	1:100	1:100	Millipore (Billerica, MA, USA)
GFAP	Mouse	1:100	1:200	Dako (Glostrup, Denmark)
MAP2	Mouse	1:500		Sigma (Saint Louis, MO, USA)
Mib1	Mouse	1:50	1:50	Dako (Glostrup, Denmark)
Nestin	Mouse	1:100	1:50	BD Transduction Laboratories (Heidelberg, Germany)
NeuN	Mouse	1:500		Chemicon International (Billerica, MA, USA)
Vimentin	Mouse	1:300		Dako (Glostrup, Denmark)

**Figure 1:**
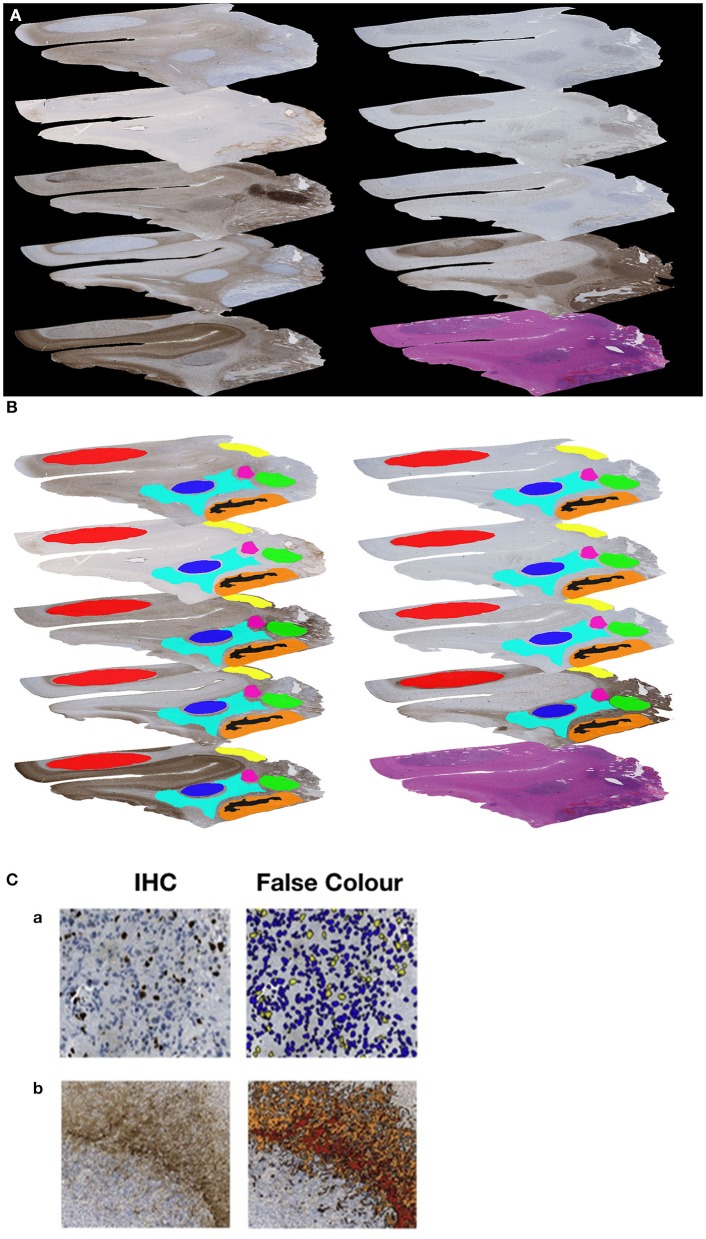
**(A)** Example of a sequence of 10 sections (nine immunohistochemical stained slides and one H&E slide). **(B)** Example of the manually generated RoIs on each of the nine immunohistochemically stained slides of the same Glioblastoma. **(C)** Example for immunohistochemical (IHC) stain and automated image analysis (False Color) for MiB-1 **(a)** and GFAP **(b)**.

As result a sequence of 10 consecutive slides for each of the 61 glioblastomas could be examined in this study. Each series consisted of one H&E and nine immunohistochemical stained slides (Figure 1A).

### Co-immunofluorescence

In selected cases, co-immunofluorescence staining was performed on additional slides using two primary antibodies. Until incubation with the first secondary antibody (dilution 1:200, 2 h) for the first epitope, steps were the same as for the immunohistochemical staining, apart from blocking with ammonium chloride (for 10 min, room temperature) to reduce autofluorescence. For amplification, slides were coated with ABC-Kit (Vector) for 1 h, followed by the combination of biotinylated Thyramid and hydrogen peroxide (1:1,000). Detection of the first target antigen was finished with the addition of Streptavidin-Alexa 555 (1:1,000). The remaining steps were continued in the dark. Incubation with the second primary antibody (Table 1), detecting the second target antigen, was performed overnight at 4°C. Then, the staining process was continued with secondary antibody, conjugated with Alexa 488 (1:200) for 2 h. Nuclei were stained with DAPI before mounting with VectaShield Mounting Medium (Vector) and applicating cover slip. Fluorescence images were captured with Axio Imager.Z2 (Zeiss) microscope equipped with a digital camera and acquisition software AxioVision Rel. 4.8 (Zeiss).

### Image Analysis

Slides were scanned (Nano Zoomer 2.0-HAT Hamamatsu) and digitized (NDP.View Hamamatsu) prior to image analysis. The quantification of the antigen presentation detected by immunohistochemistry in each selected area was performed with the object-based image analysis method, *Definiens Cognition Network Technology*. Therefore, we manually localized Regions of Interest (RoIs) to define the areas for image analysis. The selection was based on prominent antigen expression and was guided by morphological landmarks. Necrosis, vessels, larger hemorrhages, and artifacts were excluded from analysis. In total, 186 different RoIs (range 2–7 per individual tumor sample) were generated, and the corresponding areas were marked on all slides of an individual tumor to ensure comparability (Figure 1B). Using this approach, we detected and quantified (Figure 1C) the immunohistochemical visualized expression of the nine antigens in each particular region (RoI), to get a proportional measure of protein expression in the selected tumor area.

### Statistical Analysis

All statistical analyses were performed using SPSS Statistics software package.

## Results

### Correlation Analysis

In a first step, we evaluated the data (Table S1) using Spearman correlation analysis to find possible correlation (positive or negative), a linear interrelation or dependence, between the expression of individual antigens. However, considering all 186 regions only absent or weak correlations could be identified. The expression of three pairs of markers demonstrated a moderate correlation, including NeuN and MAP2 that showed a positive correlation (correlation coefficient = 0.533). Both of them further exhibited a two-dimensional dependence to EGFR (MAP2-EGFR correlation coefficient: 0.444; NeuN-EGFR correlation coefficient: 0.401). Except for these, only weak or no correlations were seen. Single values are shown in the following table (Table 2).

**Table 2:** Correlation coefficient of the nine biomarkers according to Spearman correlation analysis.

**Biomarker**	**ALDH1**	**CA-IX**	**EGFR**	**GFAP**	**MAP2**	**Mib1**	**Nestin**	**NeuN**	**Vimentin**
ALDH1	1.000	−0.102	0.041	0.108	0.113	−0.199**	−0.048	0.176*	0.010
CA-IX	−0.102	1.000	−0.122	0.003	−0.009	−0.003	0.060	−0.228**	0.224**
EGFR	0.041	−0.122	1.000	0.023	0.444**	−0.054	0.192**	0.401**	−0.256**
GFAP	0.108	0.003	0.023	1.000	−0.144	−0.219**	−0.044	−0.038	0.199**
MAP2	0.113	−0.009	0.444**	−0.144	1.000	0.231**	0.306**	0.533**	−0.042
Mib1	−0.199**	−0.003	−0.054	−0.219**	0.231**	1.000	0.243**	0.056	0.006
Nestin	−0.048	0.060	0.192**	−0.044	0.306**	0.243**	1.000	0.021	0.344**
NeuN	0.176*	−0.228**	0.401**	−0.038	0.533**	0.056	0.021	1.000	−0.180*
Vimentin	0.010	0.224**	−0.256**	0.199**	−0.042	0.006	0.344**	−0.180*	1.000

The total number of considered regions is 186. For clarity, a detailed listing of respective n-values will be omitted here. The maximum input number for some correlations is lower than 186 due to technical loss of tissue. The star symbol marks the level of significance (*p < 0.05; **p < 0.01). Bold values indicate weak correlations.

### Hierarchical Cluster Analysis

**Figure 2:**
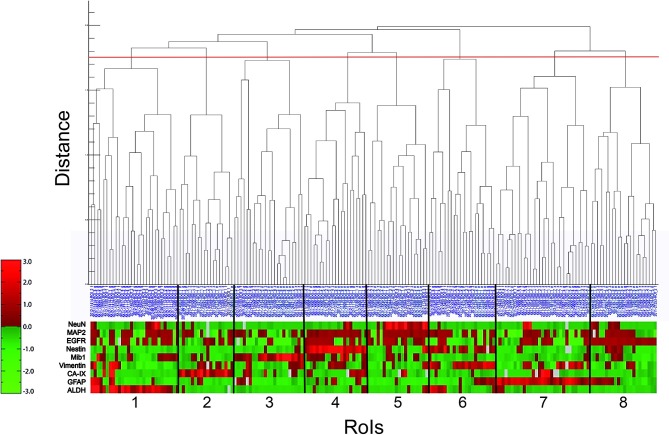
Hierarchical cluster analysis (lin_corr_norm_a3; linkage = 1). The *X*-axis lists the 186 RoIs with their blue identification number. The *Y*-axis represents the distance of item-splitting. When setting the cutoff point at 1.75 (red line), 8 clusters appear (numbered from 1 to 8). The red-green bar illustrates the immunoreactivitiy profile of each RoI with regard to the nine biomarkers (ALDH, GFAP, CA-IX, Vimentin, Mib1, Nestin, EGFR, MAP2, and NeuN). It is a relative color scale. Small values are shown in green and high values in red. Gray blocks indicate data gaps stemming from material damage during staining procedure.

**Figure 3:**
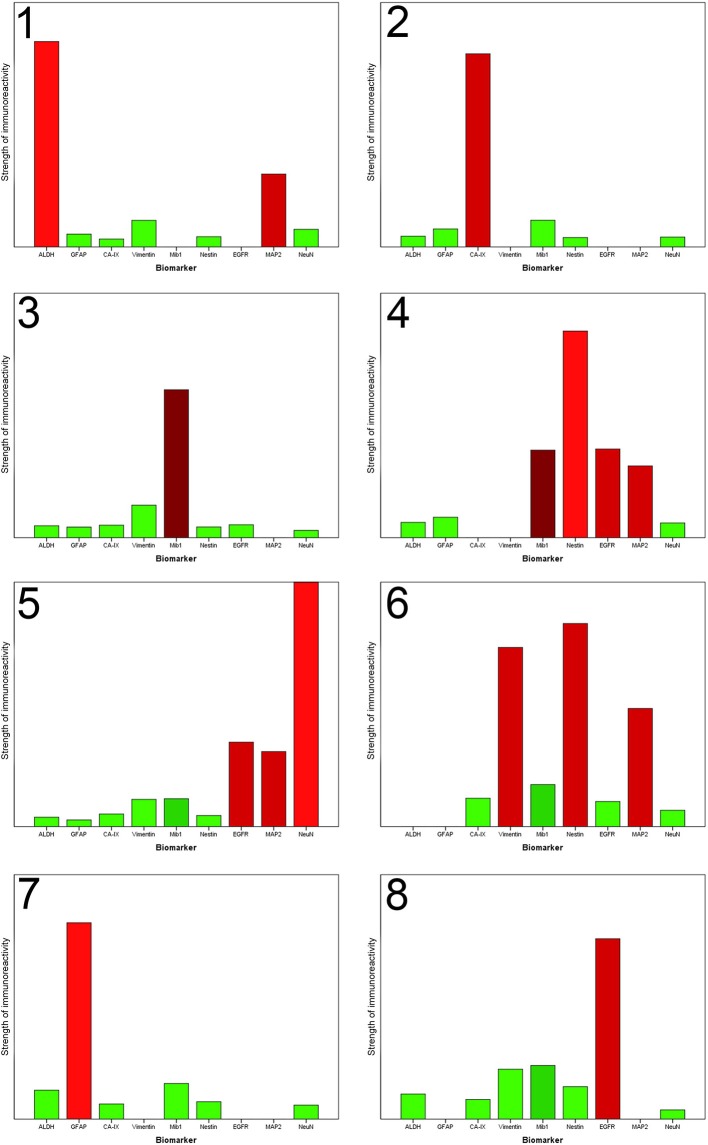
Each column chart demonstrates the homogenized representative marker profile of each newly detected cluster. The *X*-axis shows the nine biomarkers. The *Y*-axis specifies the strength of immunoreactivity. The column of NeuN in Cluster 5 is artificially shortened to obtain a better overview. A missing column indicates that the immunoreactivitiy of the biomarker in this special cluster is highly variable, that no unification is possible and the specific biomarker does not contribute to the characterization of this cluster. The coloring corresponds to the preceding illustration. Each column is colored with the in absolute terms (taking all RoIs of one cluster into account) dominant color.

Next, we performed a hierarchical cluster analysis based on a distance measure derived from linear correlation (9) of the marker profiles. Hereby, the single components of the profiles were z-transformed. As linkage criterion we applied “complete linkage” which defines the distance between two clusters as the largest distance between all intercluster pairs. We therefore looked for combinations of properties that recur throughout the whole neoplastic tissue to detect special marker profiles of all nine markers for individual RoIs. The visualized result of the hierarchical cluster analysis is shown in Figure 2. Choosing a cutoff value of 1.75, RoIs could be clustered into eight different groups of reasonable group size and homogeneity, clearly distinguished from the expression profile of the neighboring clusters. An overview of the representative marker profile of each cluster is shown in Figure 3. Each column chart demonstrates the cluster-specific combination of expressed biomarkers and therefore cluster-specific regional features.

### Expression Profiles of Individual Clusters

The results of the cluster analysis allow us a more detailed look at the specific marker profiles of each cluster (Figure 3).

#### Stem Cell and Resistance Regions

The hierarchical cluster analysis enables us to identify three clusters (number 1, 6, and 7), characterized by the expression of stem cell and resistance factors.

**Figure 4:**
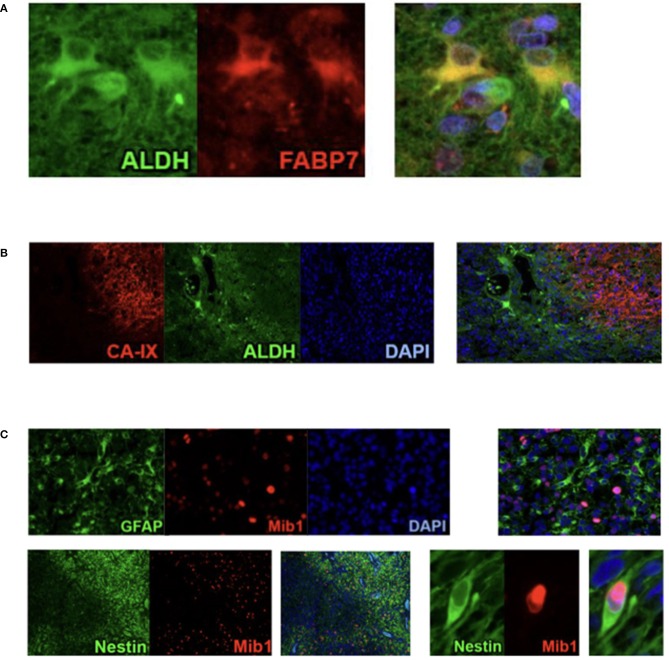
Results from Co-Immunofluorescence. **(A)** ALDH1 (green), FABP7 (red), and fusion demonstrating the stem cell characteristics of ALDH1 immunoreactive tumor cells in Stem Cell Region (ASReg, cluster 1). **(B)** Hypoxia marker CA-IX (red), ALDH1 (green), DAPI (blue), and fusion showing ALDH1 positive tumor cells located next to hypoxic regions (HReg, cluster 2). **(C)** Upper panel: GFAP (green), Mib1 (red), DAPI, and fusion demonstrating that GFAP-positive cells are very rarely proliferating; lower panel: Nestin (green), Mib1 (red) and fusion showing high percentage of Nestin-positive cells is proliferating.

Cluster 1 accumulated regions which contain numerous ALDH1-positive cells. Immunofluorescence confirmed the assumption that those cells have stem cell characteristics because it showed that ALDH1 and FABP7, which is an established biomarker for stem cells, are co-expressed in several neoplastic cells (Figure 4A). This supports our decision to name the regions of the first cluster *ALDH1-positive stem cell regions (ASReg)*.

In cluster 6, especially the intermediate filaments nestin and vimentin were found. Consistent with published studies concerning the heterogeneity of glioblastoma, in particular Phillips et al. (3) those regions are called *mesenchymal stem cell regions (MSReg)*.

Cluster 7 is dominated by the immunoreactivity of glial fibrillary acidic protein, the intermediate filaments of astrocytes, which expression is elevated under physiological conditions as a result of tissue damage and increases the resilience of the cells. Hence, we call them *astrocytic resistance regions (ARReg)*.

#### Regions of Hypoxia (HReg)

From recently published data we know that hypoxia is an inductor of resistance factors in tumor cells and neoplastic tissue (10–13). *Regions of hypoxia (HReg)* form our cluster 2. The marker profile of these regions is very impressive because only hypoxic marker CA-IX shows a high expression with only very low immunoreactivity of all remaining biomarkers. By co-immunofluorescence we established that ALDH1-positive cells as well as GFAP-positive cells are located in the fringe, directly bordering on hypoxic regions (Figure 4B).

#### Proliferative Regions (PReg)

With a medium proliferation rate of 24%, the highest tumor growth takes place in the areas of cluster 3. That is why we call these areas *highly proliferative regions (HPReg)*. NeuN expresssion as well as GFAP-immunoreactivity are barely perceptible in regions with such an amount of proliferating neoplastic cells. Co-immunofluorescene staining demonstrates on a cellular level, that Mib1-positive tumor cells express glial fibrillary acidic protein only very sporadically (Figure 4C). In regions of cluster 3, 95% of the proliferating cells are GFAP-negative. Moreover, nestin, an intermediate filament and marker for stem and progenitor cells, shows only a low immunoreactivity in highly proliferative regions.

When we look at cluster 4, however, we see that within the glioblastoma there exist other proliferative regions which combine a moderate proliferation level of 12% with a high rate of Nestin. The results of immunohistochemistry were corroborated by co-fluorescence using Mib1 and nestin, which shows high co-expression of Mib1 and nestin in a tumor area which was assigned to cluster 4 (Figure 4C). We can therefore verify, on the basis of one single tissue section, that in cluster four regions Mib1 and Nestin are co-expressed to a high degree. Consequently, the areas of cluster 4 are called *proliferative progenitor cell regions* (*PPReg*).

To conclude, in both of the proliferative clusters, GFAP and NeuN-immunoreactivity were low. Conversely, they differ in the rate of Nestin- and EGFR-expression, that were high in the *PPReg* and low in the *HPReg*.

#### Tansformed Neuronal Regions (TNReg)

The detection of neuronal antigens with a dominant NeuN-immunoreactivitiy represented in cluster 5 chraracterized the *tansformed neuronal regions (TNReg)*. The second neuronal antigen MAP2 was moderately expressed in these regions. Furthermore, EGFR-immunoreactivity was elevated in the areas of cluster number five. Resistance-indicating biomarkers (such as ALDH1, Nestin, Vimentin, and CA-IX), and GFAP, consistently showed weak expression in these areas. None of the relapsing tumors contained a TNReg.

#### Mutation Regions (MReg)

Cluster 8 regions show a strong EGFR-immunoreactivity. This could be caused by gene amplification with a consecutive overexpression of EGFR. Consequently, we call these regions *mutation regions (MReg)*. Cells of the cluster 8 proliferate by a rate of 6.6% in comparison with cluster 3 (high proliferation index and low EGFR-immunoreactivity) and cluster 4 (both, Mib1 and EGFR moderately expressed).

**Figure 5:**
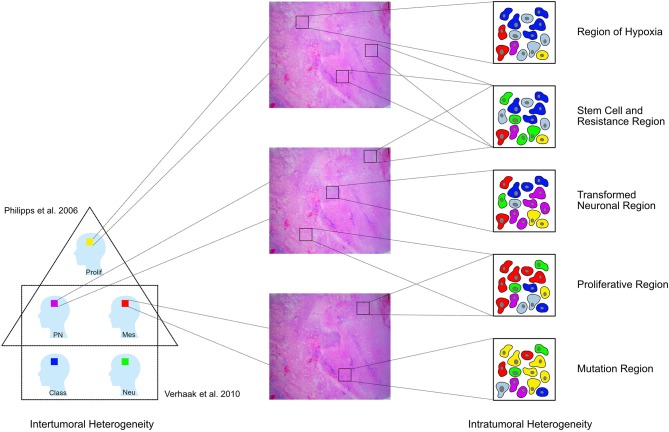
GBM have been divided into different tumoral subtypes on the basis of their molecular characteristics [Phillips et al. (3) distinguished three types proneural (PN), proliferative (Prolif), and mesenchymal (Mes); Verhaak et al. (4) classified them into four groups neural (Neu), proneural (PN), classical (Class), and mesenchymal (Mes)]. In addition to this intertumoral heterogeneity, the tumor tissue removed from one single patient also shows a heterogeneous architecture and cells with diverse features in different regions of the tumor. This study shows that the whole neoplasic tissue can be subdivided into eight clusters according to the respective immunoreactivity profile. These clusters can then be assigned to five larger pathophysiologically relevant groups [Regions of hypoxia (HReg), Stem cell and resistance regions, Tansformed neuronal regions (TNReg), Proliferative regions (PReg), and Mutation regions (MReg)].

Our data permits to classify glioblastomas into eight clusters and five upper level groups (Figure 5). After clustering, we found that, with two exceptions, all combinations of areas exist in the individual tumor tissue. For example, TNReg and HReg could be co-located in one tumor. Conversely, the proliferative areas (HPReg, PPReg) seem to exclude each other. Furthermore, we could not find an exemplary tumor with the combination of both MSReg and MReg. However, the combination of MReg and the other resistance- and stem cell regions (ASReg, ARReg) we did find.

In a next step, we inquired into whether *all* these regions (except the two mentioned above) can be found in one single tumor. To get a representative answer, we chose the two tumors of which we had the largest amount of tissue. We determined that in the tumor of a single person all of the five upper-level groups could exist together.

## Discussion

Glioblastoma is the most common primary brain tumor in adults. It is resistant to all currently available therapeutic modalities. GBM remains a fatal disease with poor prognosis, since relapse occurs regularly despite treatment. Recent investigations showed that GBM can be divided into prognostically relevant tumor subtypes, that highlight intertumoral heterogeneity (3–6). The success of this approach is, however, limited as it neglects the intratumoral heterogeneity that could be more relevant for an efficient treatment. Within a single glioblastoma different cell types with different properties and levels of resistance to therapy are located. It is most likely that current therapeutic approaches only eliminate a fraction of the tumor cells, whereas the other cell sub-populations remain intact and cause relapses. In this study, we focused on intratumoral heterogeneity to obtain additional information about the regional architecture of the tumor and analyzed different areas within individual tumors with respect to their immunoreactivity relating to nine biomarkers relevant for the biology of GBM. Consequently, this is an indirect analysis of the regional protein expression.

In a first step, we performed a correlation analysis to identify linear interrelations or interdependences between the expression of individual antigens within the tumor, but in all 186 regions only absent or weak correlations could be identified. This result underpins the presence of the remarkable intratumoral heterogeneity in GBM. In this two-dimensional model, there were almost no constant properties found all over the tumor tissue. No constant co-expression of two biomarkers could be recognized persistent in all parts of the tissue and therefore no uniform features of the entirety of all cells within the neoplastic process could be determined. This again illustrates the great importance of the intratumoral heterogeneity.

In a second step, we performed a hierarchical cluster analysis to detect the specific marker profile for every single region. Our data provides the basis to classify the GBM tissue into eight different clusters and five upper level groups, which reoccur throughout the tumor mass. Consequently, we harmonized on the regional level (Figure 5).

Three of these regions—the *transformed neuronal regions (TNReg)*, the *highly proliferative regions (HPReg)*, and the *mesenchymal stem cell regions (MSReg)*—are with regard to their characteristic marker profile similar to the three tumor subtypes described by Phillips et al. (3) (Figure 5). The *proneural GBM-subtype (PN)* was characterized by the dominant expression of NeuN and MAP2—markers used to identify cells with neuronal differentiation. The same high NeuN and MAP2-immunoreactivity is found in the *transformed neuronal regions (TNReg)* of the present study. As expected, markers for stem cells including ALDH1, nestin, and vimentin are expressed only on a very low level in those regions. A further common feature between *TNReg* and *PN* is the relatively low proliferation index. We detected not only a regional counterpart to *PN*, but also to the *proliferative subtype (Prolif)*. The latter was characterized by Phillips et al. (3) on the basis of its high proliferation index combined with a low GFAP-content in the cytoplasma of the cells. The same two attributes describe our *highly proliferative regions (HPReg)*. Using co-immunofluorescence we confirmed on a cellular level, that only five percent of the proliferating cells in those regions express GFAP. Additional homogenous properties of *Prolif* and *HPReg* are the low NeuN and vimentin immunoreaction. The third tumor subtype defined by Phillips et al. (3) is the *mesenchymale subtype (Mes)*, which is characterized by a high vimentin- and an even higher nestin-expression. The corresponding combination of biomarkers could be detected *mesenchymal stem cell regions (MSReg)* of the present study. In conclusion, we discovered that within the tissue of individual GBMs there are localized regions, that are based on their marker profile similar to previous defined tumor subtypes. *TNReg, HPReg*, and *MSReg* can exist next to each other in individual tumors. It is important to highlight that these tumor regions do not exclude each other and thus demonstrates again, that the classification of individual tumors into different tumor subtypes is limited by the existing intratumoral heterogeneity.

Consecutively the formation of tumor subtypes seems to artificially homogenize based on regions that are quantitatively predominant in the tumor sample from neurosurgical operation of one patient. But this classification is linked with the risk to overlook the minority of cell sub-populations that are perhaps resistant to the tumor-subtype-based therapy and therefore remain intact and cause a relapse.

When we consider intratumoral heterogeneity, we also have to focus on the pathophysiologically relevant *regions of hypoxia (HReg)*. With the marker profile of cluster 2, we demonstrated that in those regions where CA IX expression indicates hypoxic conditions most of the other biomarkers are almost not present. A possible explanation for this observation is a loss of antigenicity in the regions of chronic hypoxia due to proteolytic degeneration (14), reduced protein expression or increased cell death (15, 16), induced by a downregulation of metabolic processes. The late branch-off between *HReg* and *ALDH1-positive stem cell regions (ASReg)* in the hierarchical cluster analysis indicates a connection between these two regions. Indeed, as mentioned above, immunofluorescence double staining showed that ALDH1 positive cells are located in the fringe, directly bordering on hypoxic regions. In the GBM tissue, tumor stem-like cell regions are regularly found next to regions of hypoxia. The topographic relationship confirms the hypothesis that hypoxia induces the development of resistance factors in neighboring cells. The enzyme ALDH1 supports glioblastoma cells in reducing stress-induced damage including hypoxia. Hence, pathophysiologically it seems that ALDH1 is most likely elevated by hypoxic conditions (17, 18). The hypothesis that hypoxia induces the development of resistance factors is further strengthened by the fact that *astrocytic resistance regions (ARReg)* characterized by a high GFAP expression are located in the immediate vicinity of hypoxia. GFAP equips the cells with an increased mechanic stability (19) and improves its regenerative capacity. Given that hypoxia promotes the expression of proteins with cell-protective function, and proteins that enhance the motility of a cell, it is obvious that hypoxia indirectly increases the level of malignancy and the invasion potential of tumors. Consequently, hypoxia contributes also to the progression of the intratumoral heterogeneity in GBM tissue.

The *regions of hypoxia* illustrate that the regions could be pathophysiologically connected and seem to be hierarchically influenced by each other. In correspondence with findings from those studies that, addressed intertumoral heterogeneity, one may hypothesize that tumor regions identified in the present study represent areas of various steps of differentiation and development, depending on surrounding conditions and different region-specific demands on the cellular complexity, comparable with an organ anatomically constructed from cell complexes with different functions.

*TNReg* for example consists of a complex of the most differentiated cells, which are characterized by high expression of NeuN and MAP2. In regions of favorable conditions, the essential function of tumor cells is to contribute to increase the tumor mass. According to that cells in these regions proliferate and build consecutively the *proliferative regions (PReg)*. *HPReg* are characterized by a very high proliferation rate. Due to the high cell multiplication, the neoplastic cells become step by step topographically separated and are therefore at a given point exposed to different conditions. In *proliferative regions*, in which the oxygen supply is adequate proliferation can continue without limitation. In *proliferative regions*, in which the growth exceeds neovascularization, a hypo-oxygen milieu occurs (20). Prolonged hypoxia triggers the expression of CA-IX (21, 22) and forms the immunohistochemically visualized *regions of hypoxia*. While in the center of hypoxia, due to the conditions of oxygen deficiency, several biomarkers are only expressed very low in the fringe, cells that directly border on hypoxic regions acquire resistance factors (10, 11) and form *stem cell and resistance regions*. They gain resilience due to a more stable cell structure (intermediary filaments, e.g., GFAP, nestin, and vimentin), due to the improvement of coping strategies against stress (ALDH1), due to stabilization of the cell milieu (CA-IX) (23) and as a result of an increased migration capability. Such cells migrate to other regions and require an expansion of resistance features. Their main function is to ensure the perseveration of the tumor even under unfavorable conditions. The cell complexes of the *stem cell and resistance regions* are consecutively the most resilient subpopulations of the tumor even toward therapeutic measures and come to the fore in order to find an effective treatment. Standard chemo- and radiation-therapies are directed toward *proliferative regions (PReg). TNRegs* were not expressed in the tissue of relapsing tumors of the present study, whereas *Stem cell and resistance regions* were predominant in relapsing tumors. This finding is in line with the therapy- and progression-induced shift of GBM-subtype to the more aggressive one (from *PN* to *Mes*) described by Phillips et al. (3) and highlights the importance to focus on individual *stem cell and resistance regions* for fostering the development of more efficient antitumor therapies.

In conclusion, we found that GBM-tissue can be classified into eight clusters categorized to five larger pathophysiologically relevant groups, representing the intra-tumoral heterogeneity and reflecting the previously described inter-tumoral subtypes of GBM. The tumor regions seem to be pathophysiologically connected and hierarchically influenced by each other. They are areas constructed from cell complexes with different functions, depending on region-specific demands, comparable with the anatomical structure of an organ. This intratumoral heterogeneity is presumably responsible for resistance to therapy and for disease relapse. Consequently, focusing on intratumoral heterogeneity in particular *stem cell and resistance regions* seems to be an indispensable part of new therapeutic strategies and could be an attractive target of advanced antitumor therapies against GBM.

## Data Availability Statement

The datasets generated for this study are available on request to the corresponding author.

## Ethics Statement

The studies involving human participants were reviewed and approved by Ethikkommission der TUM. The patients/participants provided their written informed consent to participate in this study.

## Author Contributions

Material preparation, data collection and analysis were performed by NB, CD, AF, WB, TA, and JS. The first draft of the manuscript was written by NB. All authors commented on previous versions of the manuscript, contributed to the study conception and design, read and approved the final manuscript.

## Conflict of Interest

The authors declare that the research was conducted in the absence of any commercial or financial relationships that could be construed as a potential conflict of interest.

## Supplementary Material

Table S1 Self-explanatory.
